# *ScMT2-1-3*, a Metallothionein Gene of Sugarcane, Plays an Important Role in the Regulation of Heavy Metal Tolerance/Accumulation

**DOI:** 10.1155/2013/904769

**Published:** 2013-05-23

**Authors:** Jinlong Guo, Liping Xu, Yachun Su, Hengbo Wang, Shiwu Gao, Jingsheng Xu, Youxiong Que

**Affiliations:** Key Lab of Sugarcane Biology and Genetic Breeding, Ministry of Agriculture, Fujian Agriculture and Forestry University, Fuzhou, Fujian 350002, China

## Abstract

Plant metallothioneins (MTs), which are cysteine-rich, low-molecular-weight, and metal-binding proteins, play important roles in detoxification, metal ion homeostasis, and metal transport adjustment. In this study, a novel metallothionein gene, designated as *ScMT2-1-3* (GenBank Accession number JQ627644), was identified from sugarcane. *ScMT2-1-3* was 700 bp long, including a 240 bp open reading frame (ORF) encoding 79 amino acid residues. A His-tagged ScMT2-1-3 protein was successfully expressed in *Escherichia coli* system which had increased the host cell's tolerance to Cd^2+^, Cu^2+^, PEG, and NaCl. The expression of *ScMT2-1-3* was upregulated under Cu^2+^ stress but downregulated under Cd^2+^ stress. Real-time qPCR demonstrated that the expression levels of *ScMT2-1-3* in bud and root were over 14 times higher than those in stem and leaf, respectively. Thus, both the *E. coli* assay and sugarcane plantlets assay suggested that *ScMT2-1-3* is significantly involved in the copper detoxification and storage in the cell, but its functional mechanism in cadmium detoxification and storage in sugarcane cells needs more testification though its expressed protein could obviously increase the host *E. coli* cell's tolerance to Cd^2+^. *ScMT2-1-3* constitutes thus a new interesting candidate for elucidating the molecular mechanisms of MTs-implied plant heavy metal tolerance/accumulation and for developing sugarcane phytoremediator varieties.

## 1. Introduction

Along with the population growth and the rapid development of industrialization and urbanization, our planet is constantly subjected to various kinds of pollution damage. The heavy metal-contaminated farmland in China had already topped 20 million hectares in 2003, accounting for about 1/5 of the total cultivated area [1]. Due to the heavy metal pollution, China's annual grain production cuts in more than 1,000 million tons, and this caused a direct economic loss of about 200 billion yuans [1]. The increasing trend of pollution continued from that time and is likely to continue over the next few decades if significant remedial measures are not implemented in China.

The concept of phytoremediation was first proposed by Chaney [2] and involved the use of plants to remove pollutants from the environment or to render them harmless [3]. Phytoremediation consists of mitigating pollutant concentrations in contaminated water, soils, or air, with the ability of plants to contain, degrade, or eliminate those materials of metals, pesticides, solvents, crude oil and its derivatives, explosives and various other contaminants from the media that contain them [4]. The plant-based remediation technologies have the potential to be low cost, low impact, visually benign, and environmentally sound [5]. In recent years, there has been an increasing interest in studying the molecular mechanisms of metal accumulation and tolerance in plants [6, 7].

Sugarcane (*Saccharum* spp. L.), a major sucrose accumulator and biomass producer, is one of the most important field crops grown in the tropics and subtropics. It accounts for more than 90% of China's total sugar output at present [8]. Due to its outstanding biomass production and economic importance, sugarcane offers the potential to be a phytoremediator species, while its prospective metal accumulation and tolerance have not been fully characterized. A research on this topic was carried out by Sereno et al. [9], which showed that sugarcane could be a copper (Cu) or cadmium (Cd) phytoremediator as its plantlets were able to tolerate up to 100 *μ*M of Cu^2+^ or 500 *μ*M of Cd^2+^ in nutrient solution for 33 days without symptoms of toxicity while accumulating 45 mg Cu kg^−1^ or 451 mg Cd kg^−1^ shoot dry weight, respectively, without significant reduction in fresh weight.

Metallothioneins (MTs) are cysteine-rich, low-molecular-weight, and metal-binding proteins, which have been found in a wide variety of organisms including animals, plants, cyanobacteria, and fungi [10]. Plant MTs are extremely diverse [11] and can be classified into four subfamilies (MT1 to MT4), based on the arrangement of Cys residues [10]. Due to their ability to reversibly bind both toxic and essential metal ions, plant MTs play important roles in detoxification, metal ion homeostasis, and metal transport adjustment [10]. Consequently, the role of plant MT genes in heavy metal tolerance mechanism and phytoremediation has attracted more and more attention in recent years [7], and their ability to metal accumulation and tolerance has been demonstrated in several plants. It was shown in *Arabidopsis thaliana* that expression of *AtMT4a* gene in vegetative tissues at different developmental stages conferred increased tolerance towards Cu and Zn [12]. Transgenic *Avicennia marina* that expresses AmMT2 have been scored for enhanced tolerance to Zn, Cd, Cu, and Pb [13].

Sugarcane is one of the few species that contain genes encoding all four types of MTs [10] and one of the most potential phytoremediation species for its outstanding biomass production and high metal enrichment capability. Undoubtedly, isolation and characterization of sugarcane MT genes from sugarcane are the basis for better understanding MT gene function in heavy metal tolerance mechanism and phytoremediation. In this study, an MT2 gene, termed *ScMT2-1-3*, was successfully isolated based on large sequencing and bioinformatics analysis of the sugarcane stem full-length cDNA library. ScMT2-1-3 protein was expressed in the *E. coli *Rosetta strain, and the transgenic bacteria showed an increased tolerance both to Cd and Cu. The expression patterns of *ScMT2-1-3* in sugarcane plant were characterized in response to CdCl_2_ and CuCl_2_, and its expression levels in different sugarcane tissues were determined by real-time quantitative polymerase chain reaction (real-time qPCR).

## 2. Materials and Methods

### 2.1. Plant Materials and Treatment

Sugarcane varieties used in this study were provided kindly by the Key Laboratory of Sugarcane Biology and Genetic Breeding, Ministry of Agriculture (Fuzhou, China). Uniform tissue culture plantlets of an elite sugarcane variety FN39 were grown in 1/2 Hoagland nutrient solution for one week and then subjected to two different treatments: 500 *μ*M CdCl_2_ or 100 *μ*M CuCl_2_. The sampling times were 0 h, 3 h, 12 h, 48 h, and 72 h after the start of each treatment. All samples collected were immediately fixed in liquid nitrogen and stored in a refrigerator at −85°C until RNA extraction.

Nine healthy and consistent growing plants were randomly chosen and dug up with roots from sugarcane variety FN39 grown for 10 months. For each plant, the young root, maturing stem (internodes 4–6, 10–12, and 16–18), the leaf (+1), and all of the buds were sampled and fixed in liquid nitrogen. The collected materials were then stored in a refrigerator at −85°C until RNA extraction.

### 2.2. Molecular Cloning, Sequencing, and Bioinformatics Analysis

 The sugarcane stem full-length cDNA library was provided by the Key Laboratory of Sugarcane Biology and Genetic Breeding, Ministry of Agriculture (Fuzhou, China). *E. coli* Rosetta (DE3) and the prokaryotic expression vector pET28a were purchased from Abmart, Inc. (Shanghai, China). The restriction enzymes *Eco*R I, *Xho *I, T4 DNA ligase, *Ex*-Taq enzyme, PrimeScript RT-PCR Kit, TaKaRa LA PCR* in vitro* Cloning Kit, DNA, and protein molecular weight (MW) markers were purchased from TaKaRa (Dalian, China). HisTrap HP column was purchased from GE Healthcare Life Sciences. RQ1 RNase-Free DNase was obtained from Promega Corporation (USA), SYBR Green PCR Master Mix Kit was purchased from Applied Biosystems (USA), and the instrument used in the real-time qPCR analysis was the ABI PRISM7500 real-time PCR system.

 Large-scale sequencing and bioinformatics analysis of the full-length cDNA library of sugarcane stems were conducted as described by Guo et al. [14]. A full-length metallothionein homolog gene of sugarcane (named *ScMT2-1-3*) was identified by BLASTx (http://blast.ncbi.nlm.nih.gov/Blast.cgi) with a metallothio-2-superfamily domain (pfam01439). The open reading frame (ORF) of the full-length cDNA sequence of *ScMT2-1-3* was predicted using the ORF finder online tool from NCBI (http://www.ncbi.nlm.nih.gov/gorf/gorf.html).

The accession numbers of the chosen proteins were AhMT2a (*Arachis hypogaea*) ABA08414, AhMT2b (*A. hypogaea*) ABB05520, AtMT2a (*Arabidopsis thaliana*) NP187550, AtMT2b (*A. thaliana*) NP195858 and AAA82212, NcMT2a (*Noccaea caerulescens*) ACR46970, NcMT2b (*N. caerulescens*) ACR46962, OsMT2a (*Oryza sativa*) P94029 and AAC49627, OsMT2b (*O. sativa*) A3AZ88 and AAB18814, OsMT2c (*O. sativa*) Q5JM82 and BAA19661, PMT2a (*Populus trichocarpa × P. deltoids*) AAT02524, PMT2b (*P. trichocarpa × P. deltoids*) AAt02525, PoMT2a (*Posidonia oceanica*) CAB96155, PoMT2b (*P. oceanica*) CAB96154, SbMT2 (*Sorghum bicolor)* XP002455197, SbMT2c (*S. bicolor)* XP002439147, SmMT2a (*Salix matsudana*) ABM21761, SmMT2b (*S. matsudana*) ABM21762, SnMT2a (*Solanum nigrum*) ACF10395, SnMT2b (*S. nigrum*) ACF10396, ScMT2-1-1 (*Saccharum *complex) (the deduced amino acid sequence of CA232620/SCRUFL3063A10.g), ScMT2-1-2 (*S. *complex) AAV50043 and ABP37784, ScMT2-1-3 (*S. *complex) AFJ44225, ZmMT2-1 (*Zea mays*) NP001150795, and ZmMT2-2 (*Z. mays*) NP001147309. Alignment of putative ScMT2-1-3 protein sequence to MT2 proteins from *A. hypogaea*,* A. thaliana*,* N. caerulescens*,* O. sativa*,* P. trichocarpa × P. deltoids*,* P. oceanica*,* S. matsudana*,* S. nigrum*,* Z. mays*, *S. bicolor*,and* Saccharum *complex was performed using DNAMAN 5.2.2 software.

### 2.3. Plasmid Constructs

To study the function of *ScMT2-1-3* in prokaryotes, the *ScMT2-1-3* ORF with matched sites was amplified by PCR from the identified cDNA clone of the full-length cDNA library. The used PCR primer sequences were MT F: 5′-CGCGGATCCATG TCGTGCTGCGGAGGCAACTG-3′ and MT R: 5′-CCGCTCGAGCTTGCAGGTG CAGGGGTTGCAGC-3′ (*Bam*H I and* Xho *I sites are underlined). PCR was performed in a reaction volume of 50 *μ*L containing 5.0 *μ*L 10× PCR buffer, 4.0 *μ*L deoxynucleotide triphosphates (dNTPs) (2.5 mM), 2.0 *μ*L each of forward and reverse primers (10 *μ*M), 2.0 *μ*L plasmid DNA (100 ng), 0.25 *μ*L Ex-Taq enzyme (5 U/*μ*L), and ddH_2_O added as supplement. The PCR amplification program consisted of predenaturation for 5 min at 94°C, denaturation for 30 s at 94°C, annealing for 30 s at 55°C, extension for 30 s at 72°C for 30 cycles; and final extension for 10 min at 72°C. The *ScMT2-1-3* ORF with* Bam*H I and *Xho *I sites was subcloned into pET28a (+) (*Bam*H I-*Xho *I sites) in the *E. coli* strain Rosetta to generate the putative recombinants. A bacterial clone containing the desired recombinant plasmid was identified and validated by PCR amplification, double digestion, and sequencing, and the clone was named as pET28a-*MT2*.

### 2.4. SDS-PAGE and MALDI-TOF-TOF-MS Analysis of Prokaryotic Expression Products

The pET28a-*MT2* and empty pET28a (+) were both transformed into *E. coli* Rosetta (DE3). The single colony was inoculated into an LB medium (20 mL) containing kanamycin (50 *μ*g·mL^−1^) and chloramphenicol (170 *μ*g·mL^−1^) and incubated with 150 rpm shaking overnight at 37°C. The following day, a dilution of 1% of this overnight cultured medium was inoculated into a fresh LB medium (20 mL) containing the same concentration of kanamycin and chloramphenicol and then shake-cultured in the same conditions. When OD_600_ of the medium reached 0.4–0.6, a sample of 1.0 mL was collected as the control, and IPTG was then added to the remaining medium to a final concentration of 1.0 mM. The LB medium with pET28a-*MT2* (Rosetta) was induced for 2 h, 4 h, 6 h, and 8 h at 37°C. 100 *μ*L of the medium was collected at each time point. LB media with empty pET28a (+) (Rosetta) and blank *E. coli* Rosetta were each induced in IPTG for 8 h, after which 100 *μ*L of the cultures was collected and mixed with 25 *μ*L 5× loading buffer and then heated at 100°C for 5 min. The 10 *μ*L mixed sample was used for 12% SDS-PAGE loading. Protein molecular weight marker was used for monitoring protein separation during SDS-polyacrylamide gel electrophoresis. After electrophoresis, the gel was colored with coomassie brilliant blue and then imaged. Bio-Rad Quantity One 4.5.0 software was used to calculate the protein MW in SDS-polyacrylamide gel. At the same time, the theoretical MW of the recombinant protein was estimated using online software protein molecular weight (http://www.ualberta.ca/~stothard/javascript/protein_mw.html). The expression products were purified using HisTrap HP column, and the purified recombinant protein was analyzed by MALDI-TOF-TOF-MS for protein identification. The mass peak profiling was analyzed using online software Mascot (http://www.matrixscience.com/search_form_select.html) and MS-Digest (http://prospector.ucsf.edu/prospector/cgi-bin/msform.cgi?form=msdigest).

### 2.5. Study on the Response of *E. coli* Cells Containing Recombinant *ScMT2-1-3* Gene to Abiotic Stresses

Spot assay was performed to ascertain the response of *E. coli* Rosetta (DE3) cells transformed with recombinant plasmid (pET28a-*MT2*) or vector alone (pET28a) to Cd^2+^, Cu^2+^, PEG, and NaCl stresses. When cells grew to 0.6 (OD_600_) in LB medium, IPTG was added up to a final concentration of 1.0 mM, and then the cells were grown for further 12 h at 37°C. The cultures were diluted to 0.6 (OD_600_) and then to 10^−3^ and to 10^−4^ [15]. In group one, 10 *μ*L from each dilution was spotted on LB plates containing 100 *μ*M, 250 *μ*M, 500 *μ*M, and 750 *μ*M CdCl_2_. In group two, 10 *μ*L from each dilution was spotted on LB plates containing 50 *μ*M, 100 *μ*M, 250 *μ*M, and 500 *μ*M CuCl_2_. In group three, 10 *μ*L from each dilution was spotted on LB plates containing 250 mM, 500 mM, 750 mM, and 1000 mM NaCl. In group four, 10 *μ*L from each dilution was spotted on LB plates infiltrated with 15.0%, 20.0%, 25.0%, 30.0%, and 35.0% PEG8000 [16, 17]. All the LB plates contained 50 *μ*g·mL^−1^ kanamycin and 170 *μ*g·mL^−1^ chloramphenicol.

### 2.6. Expression Profile of *ScMT2-1-3* under Heavy Metal Stresses

Total RNA isolation was performed using the TRIzol Reagent (Invitrogen). The removal of DNA from RNA samples was realized by RQ1 RNase-Free DNase (Promega). The reverse transcription was realized by following the specifications of the PrimeScript RT reagent Kit (TaKaRa). Finally, the real-time qPCR reaction was realized by using the SYBR Green PCR Master Mix (AB).

The *25S rRNA* (BQ536525) and *GAPDH* (CA254672) genes were chosen as the internal control in the real-time qPCR analysis [18, 19], and the forward and reverse primers for *25S rRNA* were 5′-GCAGCCAAGCGTTCATA GC-3′ and 5′-CCTATTG GTGGGTGAACAATCC-3′ and for *GAPDH* were 5′-CACGGCCACTGGAAGCA-3′ and 5′-TCCTCAGGGTTCCTGATGCC-3′ [18]. From the sequence of *ScMT2-1-3*, a pair of real-time qPCR primers was designed using the Primer Express 3.0 software, and the forward and reverse primers for *ScMT2-1-3* were 5′- ACCACCCAGGCTCTCATC AT-3′ and 5′- CACTTGCACCCGTCGTTC T-3′, respectively.

The real-time qPCR reaction was realized with following conditions: 2 min at 50°C, 10 min at 95°C and then 40 cycles of 94°C for 15 s, and 60°C for 60 s. Each sample was repeated three times in the assay. When the reaction was completed, a melting curve was obtained. The 2^−ΔΔCT^ method was adopted to analyze the real-time qPCR results [20].

## 3. Results and Analysis

### 3.1. Cloning and Sequence Analysis of *ScMT2-1-3*

**Figure 1:**
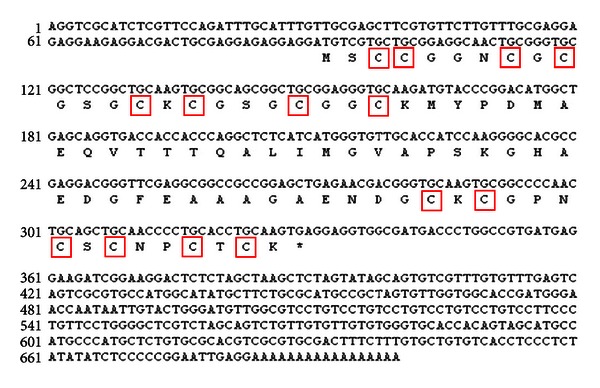
Nucleotide sequence and deduced amino acid sequence of *ScMT2-1-3*. Note: the C shows the conservative cysteine residual contained in two domains of ScMT2-1-3 with metal-binding motifs in combination among CC, CXC, and CXXC.

A full-length cDNA sequence of a metallothionein-like gene designated as *ScMT2-1-3* (GenBank Accession number JQ627644) was obtained from sugarcane by large sequencing of a stem full-length cDNA library. *ScMT2-1-3* has a full length of 700 bp, with an ORF of 240 bp, 5′ UTR (untranslated region) of 90 bp, and 3′ UTR of 370 bp (Figure 1). The deduced protein of ScMT2-1-3 was a typical plant type 2 MT-like protein which contains 14 cysteine residues distributed in two conserved cysteine-rich domains. The N-terminal domain of ScMT2-1-3 formed by eight Cys, arranged as CC, CXC, CXC and CXXC motifs, and the C-terminal domain formed by three CXC motifs, where C stands for Cys and X for variable amino acids (Figure 1).

**Figure 2:**
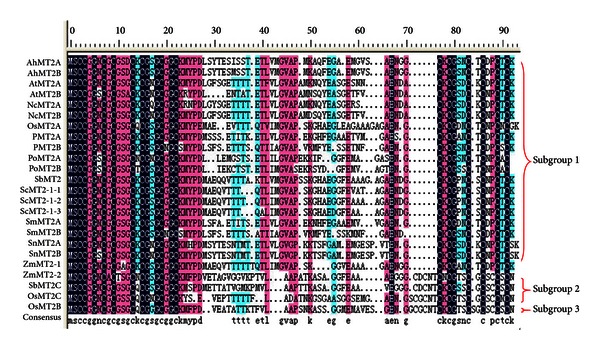
Amino acid sequence multiple alignments of MT2 proteins from different species.

ScMT2-1-3 encodes a protein which is homologous to a metallothio-2-superfamily and contains two metal-binding domains (pfam01439). Twenty-four representative MT2 protein sequences from 11 plant species, including 8 MT2 defined originally as MT2a and 8 MT2 defined originally as MT2b from the same species, respectively, were chosen for analysis of their multiple alignments in this study. ScMT2-1-3 has high homology to other plant MTs and shared 95.00% and 93.83% identity with ScMT2-1-1 and ScMT2-1-2, respectively, by their protein sequences (Figure 2, Table 1). 

### 3.2. Expression of *ScMT2-1-3* in *E. coli* Rosetta

**Figure 3:**
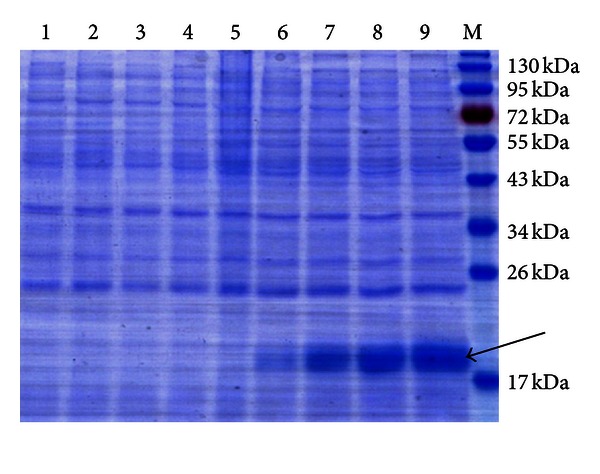
Protein expression of pET28a-*MT2* in *E. coli* Rosetta strain. M, protein marker; 1, blank without induction; 2, blank induction for 8 h; 3, control without induction; 4, control induction for 8 h; 5, pET28a-*MT2 *without induction; 6 to 9, pET28a-*MT2* induction for 2 h, 4 h, 6 h, and 8 h, respectively. IPTG-induced proteins shown by arrow.

**Figure 4:**
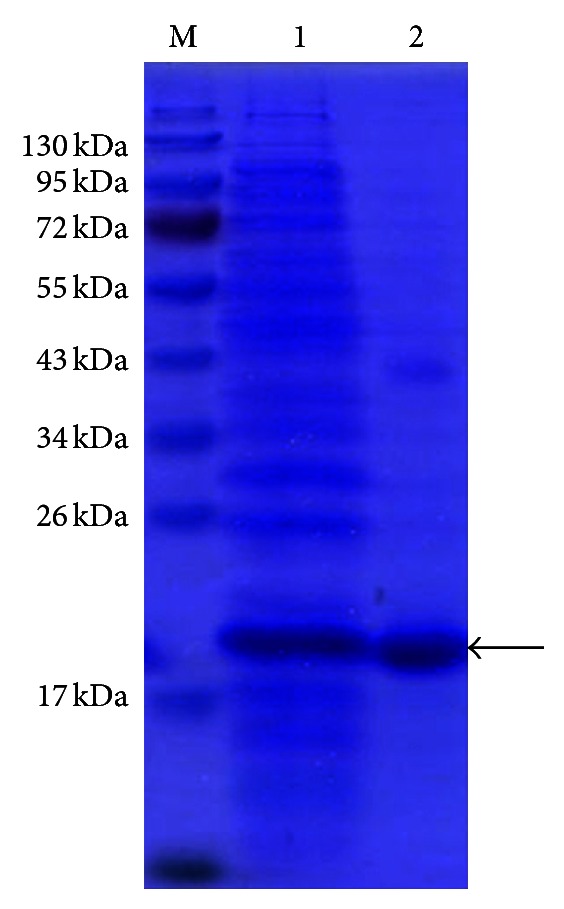
Protein purification of the recombinant protein. M, protein marker; 1, unpurified total protein; 2, Purified His-tagged-ScMT2-1-1 protein (shown by arrow).

The recombinant protein was specifically induced after 2 h of IPTG treatment and reached a maximum at 8 h (Figure 3). The expression products were purified by HisTrap HP column and showed a single band when checking an SDS gel (Figure 4). The MW of recombinant protein (His-tagged-ScMT2-1-3) was estimated to be 12.34 kDa and 13.98 kDa using the online software MS-Digest and protein molecular weight, respectively, but the protein gave a 19.01 kDa band in the SDS gel when calculated by Quantity One 4.5.0 software (Bio-Rad). We repeated the experiment and got the same results, even by changing the *E. coli* host cell for *BL21* (data not shown).

**Figure 5:**
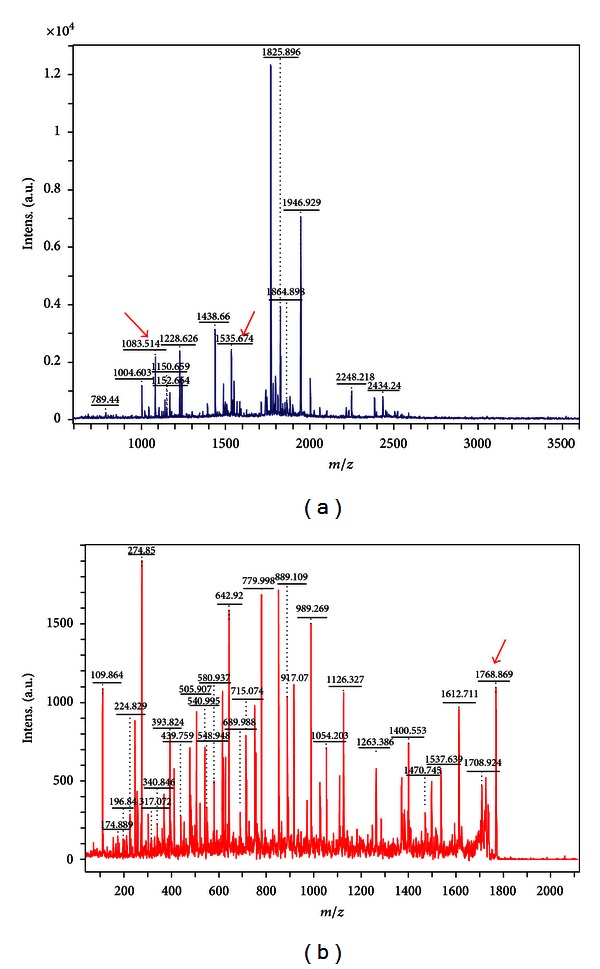
MALDI-TOF-TOF MS results of ScMT2-His.

**Figure 6:**
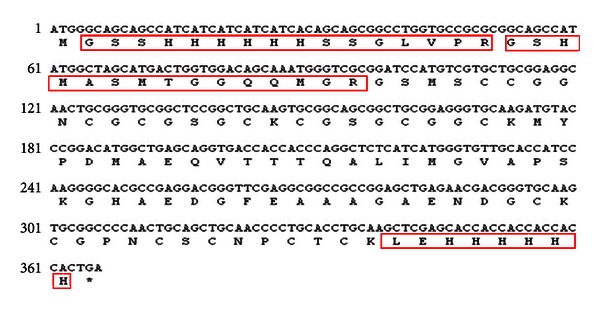
Nucleotide sequences of ScMT2-His and deduced amino acid sequences.

Further validation of the recombinant protein was realized by using MALDI-TOF-TOF MS method, and the results were analyzed using online software Mascot and MS-Digest. Three mass peaks with value of 1 083.514, 1 535.647 and 1 768.869 (Figure 5) were matched with the peptide sequences of “LEHHHHHH,” “GSHMASMTGGQQM GR,” and “GSSHHHHHHSSGLVPR,” respectively, which were partial sequences of the recombinant protein (Figure 6).

### 3.3. Overexpression of *ScMT2-1-3 *in *E. coli* Enhances Its Growth under Abiotic Stresses

**Figure 7:**
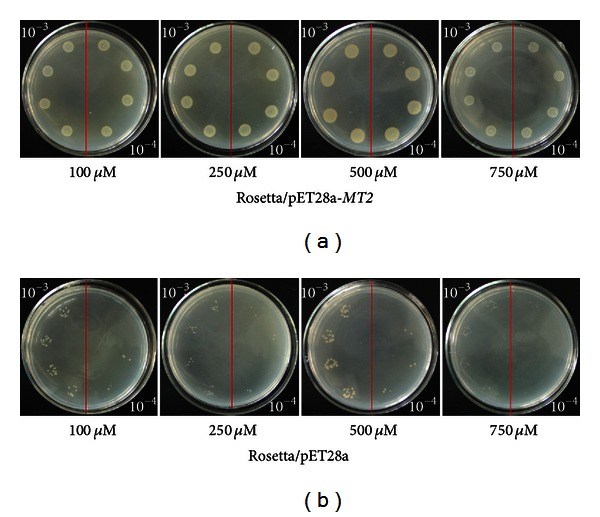
Spot assay of Rosetta/pET28a and Rosetta*/*pET28a-*MT2* on LB plates with CdCl_2_.

**Figure 8:**
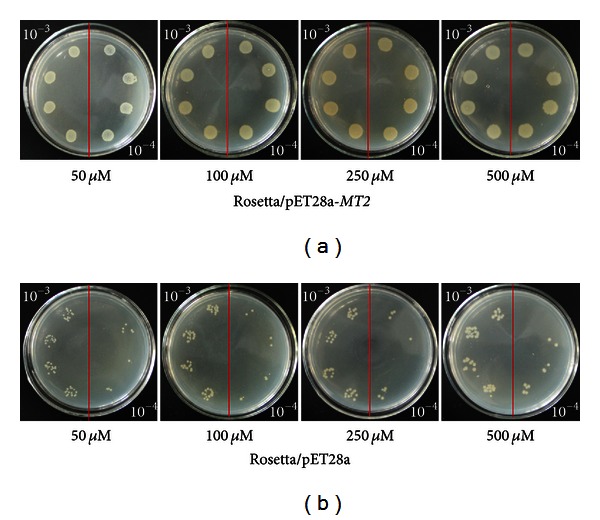
Spot assay of Rosetta/pET28a and Rosetta*/*pET28a-*MT2* on LB plates with CuCl_2_.

**Figure 9:**
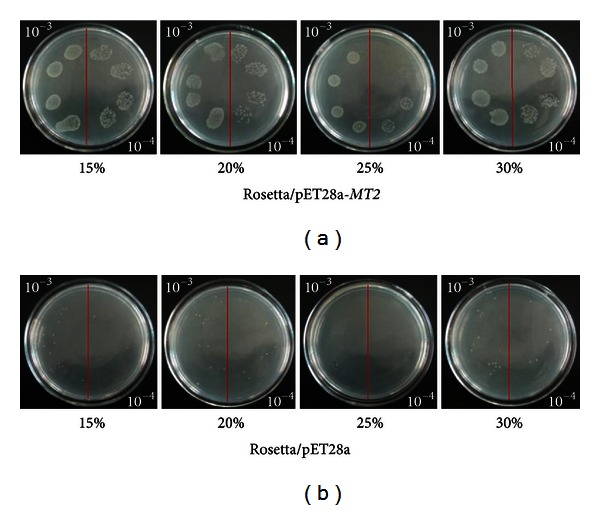
Spot assay of Rosetta/pET28a and Rosetta*/*pET28a-*MT2* on LB plates soaking with PEG.

**Figure 10:**
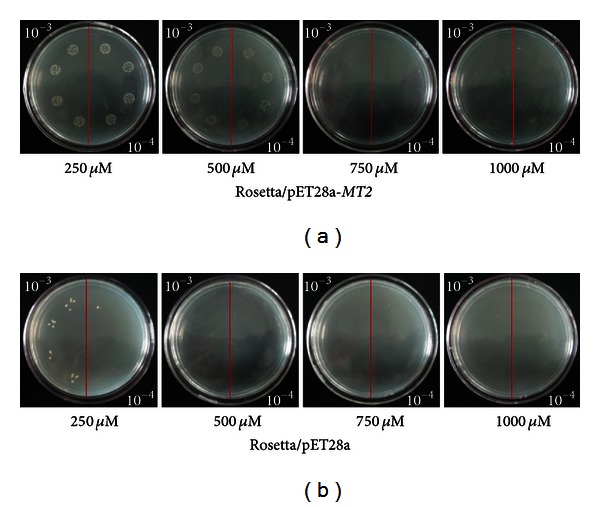
Spot assay of Rosetta/pET28a and Rosetta*/*pET28a-*MT2* on LB plates with NaCl.

Both the ScMT2-1-3 transformed and the control cells could grow in the plates containing Cd^2+^, Cu^2+^, and PEG, respectively. However, the former formed more colonies compared with the latter (Figures 7, 8, and 9). The results show that the recombinant protein enhances its growth under Cd^2+^, Cu^2+^, and PEG stresses. The growth difference was observed with the NaCl-containing LB plates after overnight culture. The ScMT2-1-3 expressed cells were able to tolerate high salt concentrations of up to 500 mM NaCl. In contrast, the growth of the control cells was obviously inhibited at 250 mM NaCl and completely inhibited at 500 mM NaCl, a lethal level for the control cells (Figure 10).

### 3.4. Tissue-Specific Expression Analysis of *ScMT2-1-3*

**Figure 11:**
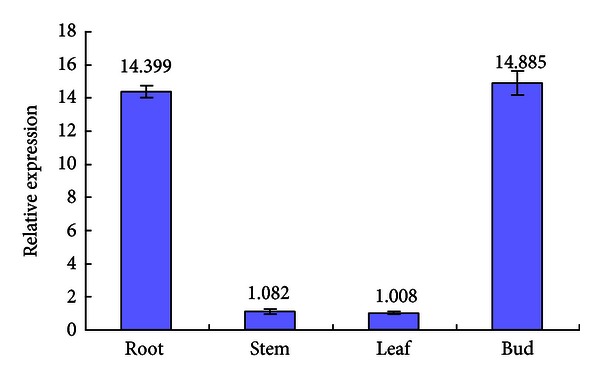
Tissue-specific expression analysis of the *ScMT2-1-3 *in sugarcane. Each value is the average of three replicate experiments ± standard error (*n* = 3).

For tissue-specific expression analysis of *ScMT2-1-3*, the sugarcane variety FN39 was used as experimental material, and the *GAPDH* gene was used as an internal control for real-time qPCR. The results showed that the *ScMT2-1-3* is highly expressed in root and bud but very lowly expressed in stem and leaf (Figure 11).

### 3.5. Expression Profile of *ScMT2-1-3* under Different Heavy Metal Stresses

**Figure 12:**
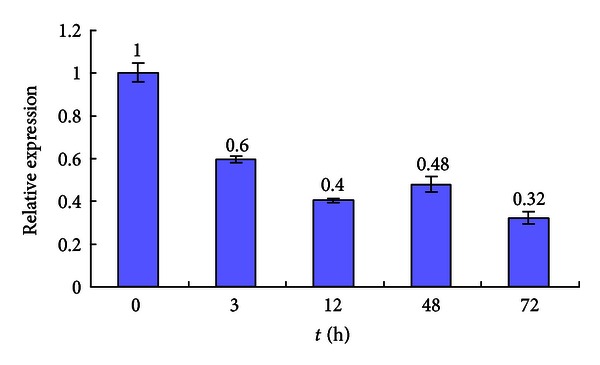
The *ScMT2-1-3* expression in sugarcane under CdCl_2_ stress. Each value is the average of three replicate experiments ± standard error (*n* = 3).

**Figure 13:**
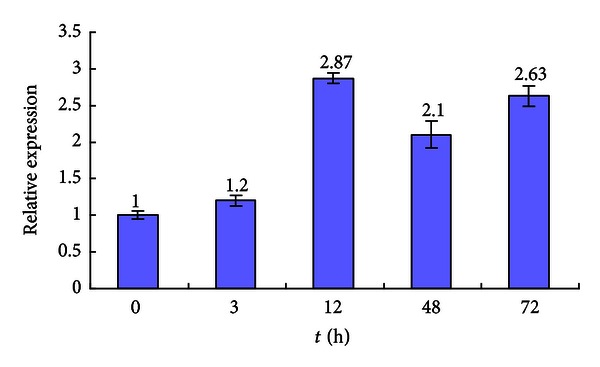
The *ScMT2-1-3 *expression in sugarcane under CuCl_2_ stress. Each value is the average of three replicate experiments ± standard error (*n* = 3).

Real-time qPCR was used to examine the expression profile of *ScMT2-1-3* on sugarcane plantlets of the variety FN39 under Cd^2+^ and Cu^2+^ stresses, respectively. The real-time qPCR results showed that the expression of *ScMT2-1-3 *was inhibited by Cd^2+^ stress which was visibly downregulated at 3 h following the treatment and maintained at a relatively lower level, compared to that of the control (Figure 12). In contrast, the expression of *ScMT2-1-3* was upregulated after Cu^2+^ treatment: first slightly increased at 3 h then obviously upregulated and reached its highest level at 12 h (2.87 times higher than that of the control), and the expression was maintained to a relatively high level (more than two times higher than that of the control) during the following examined time points (Figure 13). 

## 4. Discussion

Plant metallothionein was first discovered from soybean in 1977 [21]. Based on the sequence homology, this family of genes can be grouped into four subfamilies (Type 1 to Type 4 or MT1 to MT4) [10, 11]. Considering the large member of the plant MT family and the high sequence diversity, further subdivision should be necessary for plant MTs. In *A. thaliana*, Zhou and Goldsbrough [22] classified the MT2 proteins into two subgroups, MT2a and MT2b, according to the four codons in the central domain of AtMT2a (codons 30–33: GFSG) which are absent in AtMT2b (Figure 2). This region was shown to be highly variable among plant MTs [22]. Our analysis in Figure 2 suggests that plant MT2 proteins can be subdivided into at least 3 subgroups according to the arrangement of Cys residues (Figure 2; Table 1). This is in agreement with Wong et al. [23] who classified the rice MT2s into three subgroups termed as OsMT2a, OsMT2b, and OsMT2c, respectively. In this study, the three subgroups were termed as MT2-1, MT2-2, and MT2-3, respectively.

From the alignment of sequences, we find that the sequences of the N-terminal domain of MT2 are highly conserved (MSCCGGNCGCS) (Figure 2). MT2-1 seems to be the most abundant class among the MT2 family with typical plant MT2 Cys-rich domains pattern characterized by Cobbett and Goldsbrough [10] (Figure 2). MT2-1 contains two cysteine-rich domains separated by a spacer of approximately 40 amino acid residues (Figure 2; Table 1). The N-terminal domain contains four Cys-containing motifs. The first pair of cysteines is present as a Cys-Cys motif in amino acid positions 3 and 4 of these proteins. Two Cys-Xaa-Cys motifs are present in the center of the N-terminal cysteine-rich domain. A Cys-Xaa-Xaa-Cys motif is at the end of the N-terminal cysteine-rich domain. The C-terminal domain contains three Cys-Xaa-Cys motifs. In this study, in addition to the highly conserved N-terminal domain of MT2 (MSCCGGNCGCS-) [10], we identified an additional highly conserved motif of GVAP among this subgroup (Figure 2).

Subgroups MT2-2 and MT2-3 have the same Cys-containing motif pattern in the N-terminal, but one additional Cys-Xaa-Cys motif is present at the beginning of the C-terminal cysteine-rich domain, and one more Cys residue is randomly present between the first two Cys-Xaa-Cys motifs and the last two Cys-Xaa-Cys motifs. Moreover, the last two Cys-Xaa-Cys motifs are arranged in tandem at the end of C-terminal (Figure 2; Table 1).

Sugarcane is one of the few species which contain genes encoding all four types of MTs [10]. Of the 291, 689 ESTs in the sugarcane expressed sequence tag (SUCEST) database; a total of 849 reads (0.29%) were found to encode metallothionein-like proteins and give 55 clusters which were conceptually translated and contained the full-length protein [24]. Among the 55 clusters, 21 were related to MT2 proteins and represented 8 protein sequence variants with minor amino acid changes [24]. When ScMT2-1-1 (SCRUFL3063A10.g/CA232620) [9, 24], ScMT2-1-2 (AAV50043 and ABP37784), ScMT2-1-3 (AFJ44225), and other 7 ScMT2 proteins [24] were compared by sequence alignment, we conclude that all the MT2s in sugarcane belong to the MT2-1 subgroup and share over 93% identity in their amino acid sequences (data not shown). To date, MT2-2 and MT2-3 subgroup types have not yet been reported in sugarcane.

The expression profile of *MT2-1* genes in different organs, such as root, stem, and leaf, has been studied in several plant species. As a general evidence, the expression level of *MT2-1* genes tends to be higher in leaves than that in roots [10, 22, 25, 26]. Both *AtMT2a* (CAA44630) and *AtMT2b* (AAA82212) from *A. thaliana* were found to be highly expressed in leaves but lowly expressed in roots from mature plants [22, 25]. A similar result was obtained for *OsMT-2* gene (AAC49627) in rice [27]. In *Avicennia marina*, the level of expression of the gene *AmMT2* in leaves was found to be over 1 times higher than that in stems and 2.1 time higher than that in roots [13]. In *Hevea brasiliensis,*the gene *HbMT2* was also strongly expressed in leaves and in latex, but weakly in roots and in barks [28]. AmMT2 and HbMT2 were both classified into the subgroup of MT2-1 and shared 61.73% and 65.00% identities with ScMT2-1-3, respectively (data not shown). Based on the large-scale EST sequencing databases, the expression patterns of four types of MTs in sugarcane were investigated using 13 different sources of cDNA libraries including shoot-root zone, root, lateral bud, stem bark, stem internode, leaf, leaf roll, apex, flower, seed, callus, *in vitro* plantlet infected with *Herbaspirillum rubri* ssp. *Albicans,* and *in vitro* plantlets infected with* Gluconsugarcane diazotroficans* [24]. In general, the expression of MT2-1 in sugarcane tends to be lower in roots, higher in leaves, and so forth [9, 10, 22, 25, 26]. It is interesting to note that the expression level of *ScMT2-1-3* in roots and in buds was significantly higher (over 14 times) than those in stems and in leaves (Figure 11), never reported before.

Difficulties in identifying and isolating MTs in plants may arise from the instability of these proteins in the presence of oxygen [10]. There were few reports about expressing plant MTs in prokaryotic system, though the research on plant MTs has been carried out for decades. In some earlier studies, plant MTs have been expressed in *E. coli* as GST fusions to examine the metal-binding properties of these proteins [13, 29, 30]. Recombinant production of MTs helps to circumvent some of the problems associated with direct isolation, and expression as a GST fusion offers simple possibilities for purification, quantification, and detection [30]. GST is commonly used to create fusion proteins, and many commercially available sources of GST-tagged plasmids include a thrombin domain for cleavage of the GST tag during protein purification. GST tag has the size of 220 amino acids (roughly 26 kDa), which, compared to the low molecular mass target protein MT, is quite big. Thus, the small His-tag may be a better choice when the function of fusion protein was studied *in vivo*. In the present study, His-tag fusion protein of His-ScMT2-1-3 had successful expression in *E. coli* Rosetta (DE3), and the His-ScMT2-1-3 has an observed MW which was much greater (5.03 kDa–6.67 kDa) than that predicted by their sequences. It has been reported that the basic amino acid residues of His-tag may retard the mobility of the fusion protein bands in SDS-PAGE and cause deviation in MW determination [31]. This deviation was not observed on GST-tag fusion proteins [13, 29, 30]. Though the MW of GST-tag fusion protein GST-AtMT2a was consistent with its predicted value, the value of thrombin cleavage product after removal of GST by affinity purification was estimated at least 15 kDa in SDS-PAG which was 3 kDa greater than the predicted one [29]. Thus, similarly, the electrophoretic mobility deviation was also observed in AtMT2a [29], and the deviation can be offset by the GST-tag for its 26 kDa MW which was much greater than AtMT2a (12 kDa). We infer that this deviation might be related to the characteristics of cysteine-rich.

*AtMT2a* (X62818) gene from *A. thaliana* has been shown to be able to complement the MT-deficiency in yeast (cup1^Δ^), conferring a high level of resistance to CuSO_4_ and a moderate resistance to CdSO_4_ [22]. Guo et al. [26] have demonstrated that all four types of *A. thaliana* MTs, including *AtMT2a* (X62818) and *AtMT2b* (u11256), can offer a metal tolerance when expressed in *Saccharomyces cerevisiae*. Expression of the MT2-1 gene *PsMTa* (Z23097) from *Pisum sativum* in *E. coli* led to increased tolerance to copper and cadmium [32, 33]. Overexpression of *AmMT2* in *E. coli* BL (DE3) led to increased metal tolerance towards Zn, Cu, Pb, and Cd [13]. In a similar way, the expression of ScMT2-1-3 in *E. coli *Rosetta (DE3) enhances significantly the Cd and Cu tolerance in the present study. Furthermore, it leads to an increased tolerance to abiotic stresses of drought and salt.

Plant MTs exhibit beneficial metal-binding and induction properties which should protect these organisms from elevated levels of toxic heavy metals (such as Cd or Hg) and also affect, for example, the homeostasis of Cu and Zn, essential micronutrients for a range of plant physiological processes [10]. Some of the plant MTs' biological function of metal tolerance has been demonstrated in nonplant systems; however, MTs' *in vivo* function in plants has not yet been elucidated. We take the *MT2-1* homologous genes from various plants as samples in the following discussion. Using northern blotting technique, Zhou and Goldsbrough [22] had demonstrated that *AtMT2a* mRNA was present at a low level in *A. thaliana* 7-day-old seedlings, but the level of *AtMT2a* mRNA was increased in seedlings treated with CuSO_4_ or CdSO_4_ for 30 h. Moreover, this increase was positively correlated with metal concentration and exposure time [22]. Similarly, the regulation of *AmMT2* expression by Zn, Cu, or Pb was strongly dependent on the concentration and the time of exposure, as measured by real-time qPCR in seedlings of *A. marina* [13]. Conversely, the level of *OsMT2a* mRNA (u43530) from rice suspension cells was slightly reduced in the presence of excess Cd or Cu in the culture medium [27]. Exposures of 72 h to various concentrations of Cu, Cd, or Zn did not significantly affect the expression levels of *TcMT2* in shoots of 5-week-old *Thlaspi caerulescens* seedlings [11]. A subsequent study of *AtMT2* on 7-day-old *A. thaliana* seedlings had demonstrated that *AtMT2a* is strongly induced by CuSO_4_ (50 *μ*M), whereas *AtMT2b* remains insensitive to the same condition [22]. It seems that *TcMT2* and *AtMT2b* genes are expressed constitutively in some plant organs or tissues [11, 22]. Further study by real-time qPCR showed that although copper treatment (40 *μ*M CuCl_2_) failed to cause a significant increase in the expression of *AtMT2a* in roots and in primary leaves of 6.5-day-old seedlings, the copper-induced increase in *AtMT2a* mRNA was restricted to the cotyledons and, to a lesser extent, the hypocotyl [34]. Consistent with the results of García-Hernández et al. [34], RNA blots showed that the levels of *AtMT2a* and *AtMT2b* RNA increased after Cu treatment, but not for every gene in every tissue [25]. The Cu treatment increased the mRNA expression of *AtMT2b* in roots and *AtMT2a* in leaves [25]. Thus, they suggested that the plant MTs have distinct functions in heavy metal homeostasis [25]. It should be stressed that although it is believed that plant MTs could play an important role in heavy metal tolerance mechanism and phytoremediation, the precise function of these MTs in plant tolerance to abiotic stresses is still not clear because of the lack of information.

It has been reported that sugarcane plantlets were able to tolerate up to 100 mM of Cu or 500 mM of Cd in nutrient solution for 33 days while accumulating 45 mg Cu per kg or 451 mg Cd per kg shoot dry weight [9]. Using RNA blot, the expression patterns of sugarcane MT genes, including *ScMT2-1-1*, in shoots and in roots, were analyzed under increasing concentrations of copper and cadmium [9]. Increasing Cu concentration had little or no effect in modulating the expressions of MT genes, while an apparent minor modulation of some of the MT genes was detected in Cd treatments which presented a minor downregulation in 33 days Cd treatment samples. In this study, we showed that the level of *ScMT2-1-3* expression in Cd-treated plantlets decreased steadily 3 h following the treatment and maintained a low expression level up to 72 h. This result was in agreement with Sereno et al. [9], who inferred that cadmium tolerance and accumulation in sugarcane might derive from other mechanisms. We infer that not ScMT2-1-3 but other member(s) of metallothioneins or phytochelatins play a key role in cadmium detoxification and homeostasis in sugarcane, although ScMT2-1-3 has the ability of imparting Cd tolerance when expressed in *E. coli*. Clearly different from *ScMT2-1-1* observed by Sereno et al. [9], steadily the increased expression level of *ScMT2-1-3* began to be observed at 3 h after Cu treatment, and the expression maintained 2 times higher than the control during the time examined. Thus, both the *E. coli* assay and sugarcane plantlets assays suggested that *ScMT2-1-3* is significantly involved in the copper detoxification and storage in the cell. The differential expression patterns of *ScMT2-1* in response to Cd or Cu exposure, observed by Sereno et al. [9], and this study, suggested that the members of *ScMT2-1* genes may have diverse roles or functions.

According to their chemical and physical properties, two different molecular mechanisms of heavy metal toxicity caused by copper and cadmium have been reported: (a) production of reactive oxygen species by autoxidation and Fenton reaction, which is typical for transition metal copper [35, 36]; (b) blocking of essential functional groups in biomolecules, which is well documented for nonredox-reactive heavy metal cadmium [37]. On the one hand, the different expression pattern of *ScMT2-1-3 *may suggest different molecular mechanisms of heavy metal toxicity caused by Cd^2+^ and Cu^2+^ according to their chemical and physical properties. On the other hand, the up-regulation of *ScMT2-1-3 *under the stress of Cu^2+^ indicated that this gene is significantly involved in the copper detoxification and storage in sugarcane cells, while the downregulation of *ScMT2-1-3 *under the stress of Cd^2+^ implied that its functional mechanism in cadmium detoxification and storage in sugarcane cells needs more testification. 

## 5. Conclusions

In conclusion, we reported here a new member of plant type 2 metallothionein subfamily, termed as *ScMT2-1-3 *identified in sugarcane. We demonstrated that the expression of ScMT2-1-3 in *E. coli *can significantly enhance the tolerance to abiotic stresses such as heavy metal (copper and cadmium), droughtly and salt stresses. In contrast with the previous, reported MTs in sugarcane, *ScMT2-1-3* has a distinct expression pattern in response to copper and cadmium treatments: highly expressed in root and bud but lowly expressed in stem and leaf; more interestingly, its expression is clearly upregulated by copper and downregulated by cadmium in sugarcane. These results, taken together, showed that *ScMT2-1-3* was involved in the response to copper stresses, while cadmium tolerance and accumulation in sugarcane might derive from other mechanisms, maybe compensation mechanisms though this deduction needs more testification. *ScMT2-1-3* constitutes thus a new interesting candidate for elucidating the molecular mechanisms of MTs-implied plant heavy metal tolerance/accumulation and for developing sugarcane phytoremediator varieties.

## Figures and Tables

**Table 1:** Classification of MT2s of plants.

Subgroup	Feature of Cys-rich domains at amino- and carboxy-terminal regions
MT2-1	CCXXXCXCXXXCXCXXXCXXC......CXCXXXCXCXXCXC
MT2-2	CCXXXCXCXXXCXCXXXCXXC......CXCXXCXCXXXCXCXCCXC
MT2-3	CCXXXCXCXXXCXCXXXCXXC......CXCXXCXCXXXCXXCXCCXC

Note: “C” represents a Cys residue, X represents an amino acid residue other than Cys, and “......” represents the intermediate region between the two Cys-rich domains.

## References

[1] Gu JG, Zhou QX, Wang X (2003). Reused path of heavy metal pollution in soils and its research advance. *Journal of Basic Science and Engineering*.

[2] Chaney RL, Parr JF, Marsh PB, Kla JM Plant uptake of inorganic waste constituents-. *Land Treatment of Hazardous Wastes*.

[3] Raskin I, Smith RD, Salt DE (1997). Phytoremediation of metals: using plants to remove pollutants from the environment. *Current Opinion in Biotechnology*.

[4] Flathman PE, Lanza GR (1998). Phytoremediation: current views on an emerging green technology. *Journal of Soil Contamination*.

[5] Cunningham SD, Ow DW (1996). Promises and prospects of phytoremediation. *Plant Physiology*.

[6] Verbruggen N, Hermans C, Schat H (2009). Molecular mechanisms of metal hyperaccumulation in plants. *New Phytologist*.

[7] Hossain MA, Piyatida P, Teixeira da Silva JA, Fujita M (2012). Molecular mechanism of heavy metal toxicity and tolerance in plants: central role of glutathione in detoxification of reactive oxygen species and methylglyoxal and in heavy metal chelation. *Journal of Botany*.

[8] Wu MG, Lin YQ, Zhang H (2010). Research status and prospect on industrial standard of sugarcane in China. *Subtropical Agriculture Research*.

[9] Sereno ML, Almeida RS, Nishimura DS, Figueira A (2007). Response of sugarcane to increasing concentrations of copper and cadmium and expression of metallothionein genes. *Journal of Plant Physiology*.

[10] Cobbett C, Goldsbrough P (2002). Phytochelatins and metallothioneins: roles in heavy metal detoxification and homeostasis. *Annual Review of Plant Biology*.

[11] Roosens NH, Leplae R, Bernard C, Verbruggen N (2005). Variations in plant metallothioneins: the heavy metal hyperaccumulator *Thlaspi caerulescens* as a study case. *Planta*.

[12] Rodríguez-Llorente ID (2010). Epxression of the seed-specific metallothionein mt4a in plant vegetative tissues increases Cu and Zn tolerance. *Plant Science*.

[13] Huang GY, Wang YS (2010). Expression and characterization analysis of type 2 metallothionein from grey mangrove species (*Avicennia marina*) in response to metal stress. *Aquatic Toxicology*.

[14] Guo JL, Que YX, Liu JX, Zheng YF, Chen RK, Xu LP (2009). Construction of full-length cDNA library for sugarcane stem by optimized oligo-capping. *Chinese Journal of Troical Crops*.

[15] Gupta K, Agarwal PK, Reddy MK, Jha B (2010). SbDREB2A, an A-2 type DREB transcription factor from extreme halophyte *Salicornia brachiata* confers abiotic stress tolerance in *Escherichia coli*. *Plant Cell Reports*.

[16] van der Weele CM, Spollen WG, Sharp RE, Baskin TI (2000). Growth of *Arabidopsis thaliana* seedlings under water deficit studied by control of water potential in nutrient-agar media. *Journal of Experimental Botany*.

[17] Zhang LJ, Huan LJ, Ruan YY, Guan YX (2004). Application of polyethylene glycol in the study of plant osmotic stress physiology. *Plant Physiology Communications*.

[18] Iskandar HM, Simpson RS, Casu RE, Bonnett GD, Maclean DJ, Manners JM (2004). Comparison of reference genes for quantitative real-time polymerase chain reaction analysis of gene expression in sugarcane. *Plant Molecular Biology Reporter*.

[19] Que YX, Xu LP, Xu JS, Zhang JS, Zhang MQ, Chen RK (2009). Selection of control genes in real-time qPCR analysis of gene expression in sugarcane. *Chinse Journal of Tropical Crops*.

[20] Livak KJ, Schmittgen TD (2001). Analysis of relative gene expression data using real-time quantitative PCR and the 2^-ΔΔ^CT method. *Methods*.

[21] Bartolf M, Brennan E, Price CA (1980). Partial characterization of a cadmium-binding protein from the roots of cadmium-treated tomato. *Plant Physiology*.

[22] Zhou J, Goldsbrough PB (1995). Structure, organization and expression of the metallothionein gene family in *Arabidopsis*. *Molecular and General Genetics*.

[23] Wong HL, Sakamoto T, Kawasaki T, Umemura K, Shimamoto K (2004). Down-regulation of metallothionein, a reactive oxygen scavenger, by the small GTPase OsRac1 in rice. *Plant Physiology*.

[24] Figueira A, Kido EA, Almeida RS (2001). Identifying sugarcane expressed sequences associated with nutrient transporters and peptide metal chelators. *Genetics and Molecular Biology*.

[25] Guo WJ, Bundithya W, Goldsbrough PB (2003). Characterization of the *Arabidopsis* metallothionein gene family: tissue-specific expression and induction during senescence and in response to copper. *New Phytologist*.

[26] Guo WJ, Meetam M, Goldsbrough PB (2008). Examining the specific contributions of individual *Arabidopsis* metallothioneins to copper distribution and metal tolerance. *Plant Physiology*.

[27] Hsieh HM, Liu WK, Chang A, Huang PC (1996). RNA expression patterns of a type 2 metallothionein-like gene from rice. *Plant Molecular Biology*.

[28] Zhu J, Zhang Q, Wu R, Zhang Z (2010). *HbMT2*, an ethephon-induced metallothionein gene from *Hevea brasiliensis* responds to H_2_O_2_ stress. *Plant Physiology and Biochemistry*.

[29] Murphy A, Zhou J, Goldsbrough PB, Taiz L (1997). Purification and immunological identification of metallothioneins 1 and 2 from *Arabidopsk thaliana*. *Plant Physiology*.

[30] Bilecen K, Ozturk UH, Duru AD (2005). *Triticum durum* metallothionein: isolation of the gene and structural characterization of the protein using solution scattering and molecular modeling. *Journal of Biological Chemistry*.

[31] Tang WH, Zhang JL, Wang ZY, Hong MM (2000). The cause of deviation made in determining the molecular weight of His-tag fusion proteins by SDS-PAGE. *Acta Phytophysio-Logica Sinica*.

[32] Tommey AM, Shi J, Lindsay WP, Urwin PE, Robinson NJ (1991). Expression of the pea gene PsMT(A) in *E. coli*: metal binding properties of the expressed protein. *FEBS Letters*.

[33] Evans KM, Gatehouse JA, Lindsay WP, Shi J, Tommey AM, Robinson NJ (1992). Expression of the pea metallothionein-like gene PsMTA in *Escherichia coli* and *Arabidopsis thaliana* and analysis of trace metal ion accumulation: implications for PsMTA function. *Plant Molecular Biology*.

[34] García-Hernández M, Murphy A, Taiz L (1998). Metallothioneins 1 and 2 have distinct but overlapping expression patterns in *Arabidopsis*. *Plant Physiology*.

[35] Li Y, Trush MA (1993). DNA damage resulting from the oxidation of hydroquinone by copper: role for a Cu(II)/Cu(I)) redox cycle and reactive oxygen generation. *Carcinogenesis*.

[36] Li Y, Trush MA (1993). Oxidation of hydroquinone by copper: chemical mechanism and biological effects. *Archives of Biochemistry and Biophysics*.

[37] Rivetta A, Negrini N, Cocucci M (1997). Involvement of Ca^2+^-calmodulin in Cd^2+^ toxicity during the early phases of radish (*Raphanus sativus* L.) seed germination. *Plant, Cell and Environment*.

